# Circular RNAs in diabetes mellitus and its complications

**DOI:** 10.3389/fendo.2022.885650

**Published:** 2022-08-01

**Authors:** Wenqi Fan, Haipeng Pang, Zhiguo Xie, Gan Huang, Zhiguang Zhou

**Affiliations:** National Clinical Research Center for Metabolic Diseases, Key Laboratory of Diabetes Immunology (Central South University), Ministry of Education, and Department of Metabolism and Endocrinology, The Second Xiangya Hospital of Central South University, Changsha, China

**Keywords:** CircRNAs, type 1 diabetes mellitus, type 2 diabetes mellitus, gestational diabetes mellitus, diabetes-associated complications

## Abstract

Diabetes mellitus (DM) is an endocrine disorder characterized by a relative or absolute lack of insulin due to the dysfunction or destruction of β-cells. DM is one of the fastest growing challenges to global health in the 21^st^ century and places a tremendous burden on affected individuals and their families and countries. Although insulin and antidiabetic drugs have been used to treat DM, a radical cure for the disease is unavailable. The pathogenesis of DM remains unclear. Emerging roles of circular RNAs (circRNAs) in DM have become a subject of global research. CircRNAs have been verified to participate in the onset and progression of DM, implying their potential roles as novel biomarkers and treatment tools. In the present review, we briefly introduce the characteristics of circRNAs. Next, we focus on specific roles of circRNAs in type 1 diabetes mellitus, type 2 diabetes mellitus, gestational diabetes mellitus and diabetes-associated complications.

## Introduction

Diabetes mellitus (DM) encompasses several heterogeneous disorders that occur when a lack of insulin or insulin resistance evokes a high level of blood glucose. Long-term hyperglycemia undermines the organs of the body, contributing to a group of DM-associated complications. The common types of DM include type 1 diabetes mellitus (T1DM), type 2 diabetes mellitus (T2DM), and gestational diabetes mellitus (GDM). Additionally, specific types of diabetes have been identified, such as monogenic diabetes syndromes, diseases of the exocrine pancreas, and drug- or chemical-induced diabetes (1). However, these specific types of diabetes are not included in this review because they are rare. Prevalence of DM is alarming. According to the latest International Diabetes Federation (IDF) Diabetes Atlas, the number of people aged 20-79 years with DM was estimated to be 536.6 million (10.5% of the global population) in 2021, and this number is predicted to reach 783.2 million (12.2%) by 2045 (2). Globally, almost one in two (44.7%; 239.7 million) adults (20–79 years of age) with DM are living with undiagnosed DM (3). Late diagnosis of DM corresponds to the greater risk of developing complications. In summary, increasing number of patients with DM and its serious complications, such as diabetic retinopathy (DR), diabetic nephropathy (DN), and diabetic cardiomyopathy (DC) (4), exerts an undue pressure on global health. The etiology of DM remains obscure despite extensive research efforts. However, as long as a patient is diagnosed early and appropriate therapy is applied, DM and its complications can be adequately controlled. Thus, a biomarker with high sensitivity and high specificity and an effective method of therapy are imperative.

In recent years, the associations between circular RNAs (circRNAs) and DM have been demonstrated (5–8). CircRNAs are endogenous biomolecules with a structure of covalently closed transcript loops. CircRNAs are expressed in various organisms, from fruit flies and worms to mice and humans (7). CircRNAs can serve as the sponges for specific miRNAs to further regulate the expression levels of the target genes. Advancement of RNA sequencing technology and bioinformatics-based prediction resulted in identification of an increasing number of circRNAs and their biological functions (6, 8–10).

**Figure 1:**
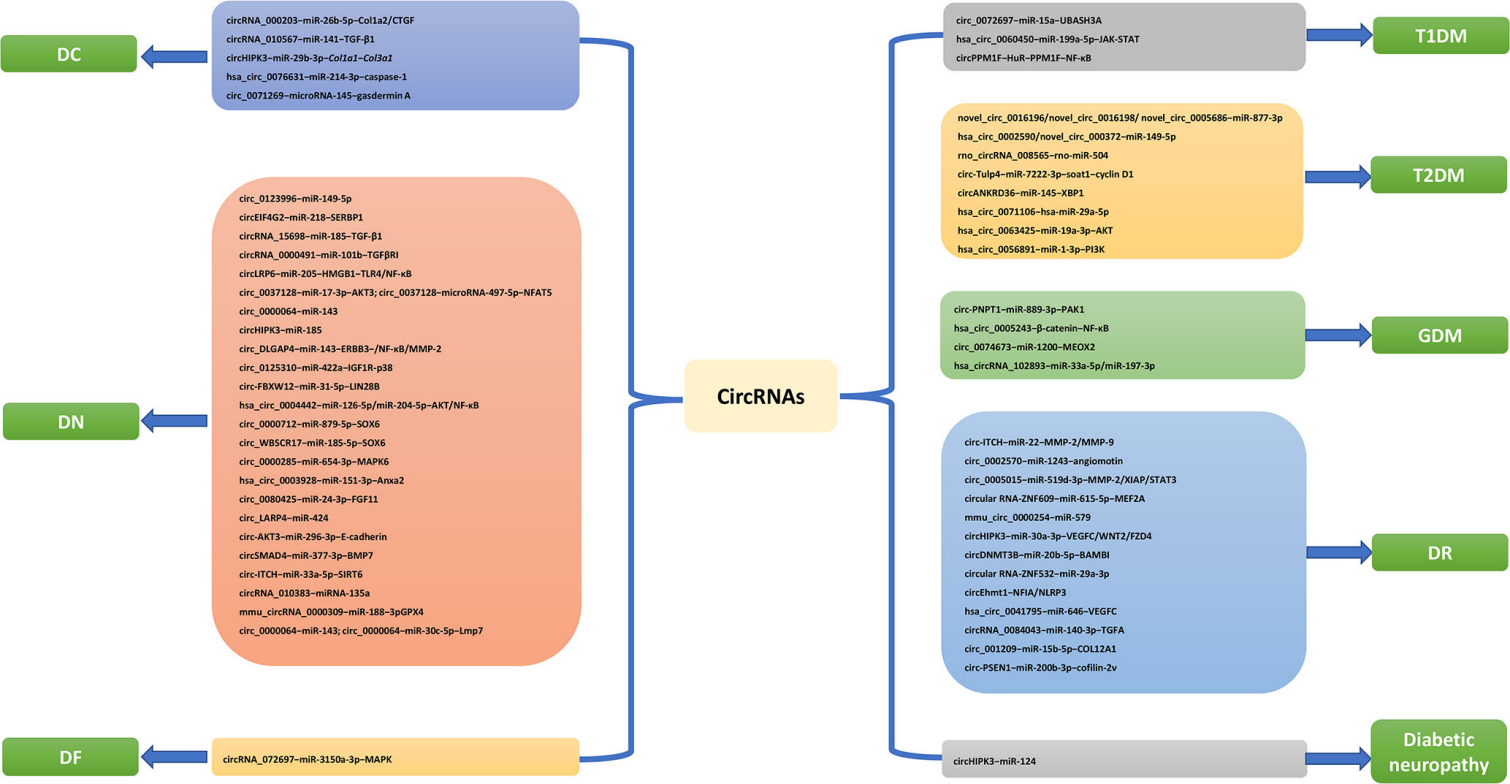
CircRNA pathways related to DM and its complications.

Current studies have reported the effects of circRNAs on DM (**Figure 1**). For example, the levels of circHIPK3 and ciRS-7/CDR1as are decreased in the islets in a DM mouse model, and this decrease may disrupt β-cell function, including insulin secretion and proliferation (5). Additionally, a study demonstrated an enriched circRNA profile in human pancreatic islets from the donors with T2DM. CircCIRBP was shown to be associated with the insulin secretory index in isolated human islets, and the expression of circCIRBP and circRPH3AL is altered after the contents of fatty acids are elevated in EndoC-βH1 cells. This evidence revealed that understanding of the actions of circRNAs may elucidate the etiology and progression of DM. Additionally, circCAMSAP1 is abundant in human pancreatic islets and is expressed in peripheral blood (6), uncovering the potential use of circRNAs as biomarkers because of their easy availability. Furthermore, circ-TGFBR3, a highly β-cell-selective circRNA, encodes the transforming growth factor (TGF)-β type III receptor and acts mainly by binding with miR-29a-5p and miR-874-3p (11). A recent study used small molecule inhibitors of the TGF-β pathway to demonstrate that the suppression of TGF-β signaling facilitates human pancreatic β-cell replication in human islets transplanted into nonobese diabetic (NOD) mice (12). The destruction of β-cells is the key factor in the progression of DM; in contrast, regeneration of β-cells *via* self-replication has high potential to restore pancreatic islet function, offering new avenues for DM therapy.

In conclusion, these findings revealed that circRNAs are not only involved in the etiology and progression of DM but also may be used as biomarkers or even play a crucial role in regenerative medicine. Therefore, many very recent studies have focused on various circRNAs and their relevant functions under diabetic conditions with the hope of using these circRNAs to predict and cure DM. However, additional studies are warranted before clinical applications.

## Characteristics of circRNAs

In the past, noncoding RNAs (ncRNAs) were regarded as transcriptional noise; however, increasing evidence identified ncRNAs as regulators of diverse biological processes (13). Based on their functions, ncRNAs can be divided into structural or housekeeping ncRNAs (e.g., transfer RNAs, ribosomal RNAs, and small nucleolar RNAs) and regulatory RNAs (14). Regulatory RNAs mainly comprise small interfering RNAs (siRNAs), piwi-RNAs (piRNAs), microRNAs (miRNAs), long noncoding RNAs (lncRNAs), and circRNAs. Previous studies were predominantly focused on miRNAs and lncRNAs. Recently, circRNAs have gained increasing attention of the researchers because of their potential as novel biomarkers for early detection of pathological states and as therapeutic targets to ameliorate the clinical burden of the diseases.

**Figure 2:**
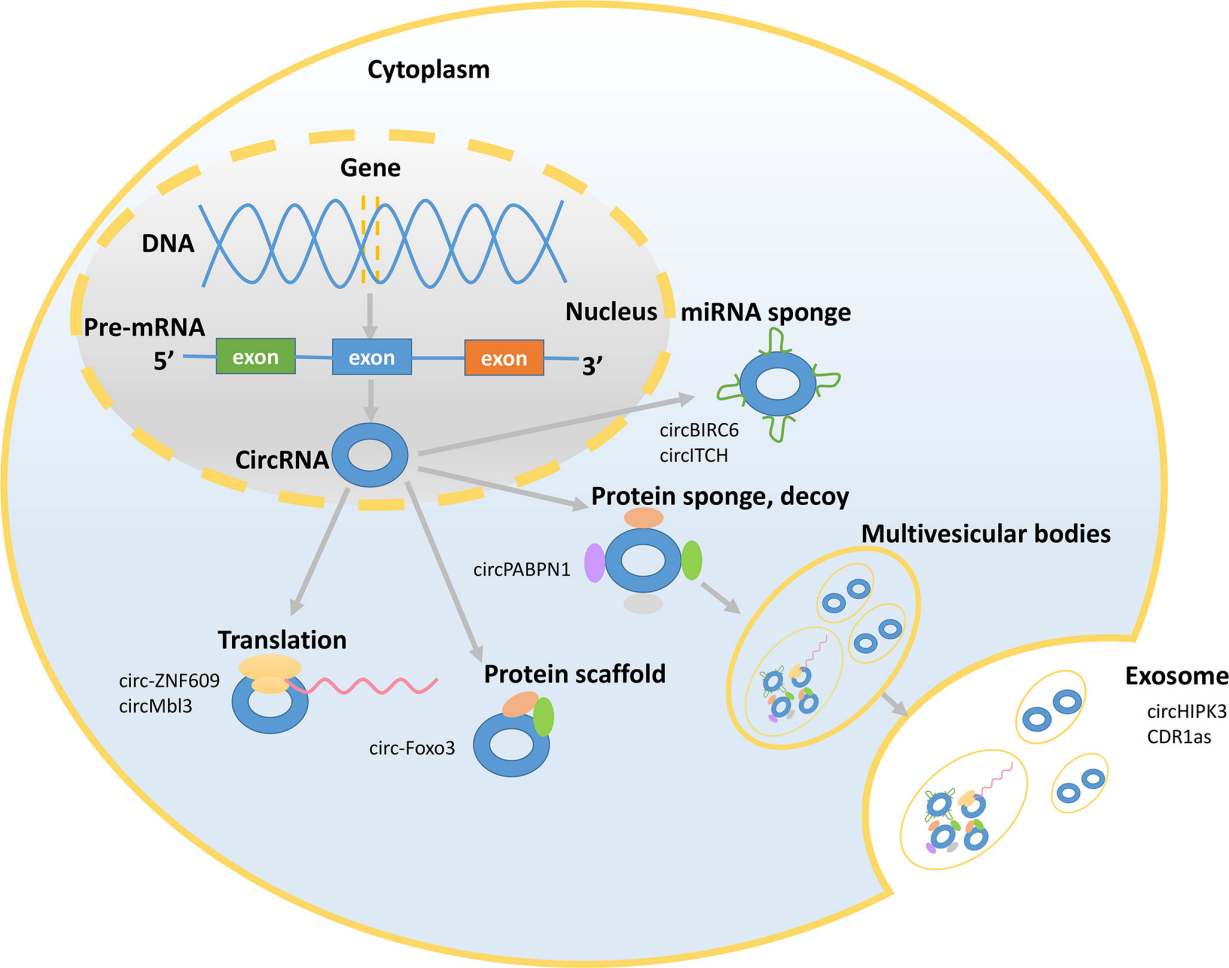
Mechanisms underlying circRNA functions. The picture shows that circRNAs exert their functions in the nucleus and cytoplasm and presents some representative circRNAs. CircRNAs can act as miRNA sponges and bind to RNA-binding proteins (RBPs) and can be translated and sorted to the exosomes.

Although the circular nature of circRNAs renders their detection and functional annotation challenging, progress in high-throughput RNA sequencing technology and computational algorithms has enabled identification of many endogenous circRNAs in various tissues. These RNA species are covalently closed single-stranded transcripts derived by back-splicing of precursor messenger RNA (pre-mRNA) in eukaryotes (**Figure 2**) (7). According to their structures, circRNAs can be classified as exon–intron circRNAs (EIciRNAs), intronic circRNAs (ciRNAs), and exonic circRNAs (ecircRNAs) (15). Compared with their linear counterparts (4.0−7.4 h), circRNAs have longer half-lives (18.8–23.7 h) because they lack a 5’ cap and 3’ tail, protecting the molecules against RNase degradation (16). In addition to stability, age-based accumulation trends are another notable feature of circRNAs (17). Consistent with these trends, the number of circRNAs at a specific stage reflects the characteristics of host cells, and this property may be helpful for disease prediction.

CircRNAs exert their functions *via* various mechanisms (**Figure 2**). First, circRNAs can act as miRNA sponges to influence the expression of the target genes by suppressing miRNA activity (18). CircRNAs compete with parental genes for the miRNA-binding sites given that circRNAs have the reverse sequence of the parental genes. A single circRNA that functions as a competitive endogenous RNA is able to regulate one or multiple miRNAs *via* its miRNA-binding sites. Moreover, the miRNA-binding sites of circRNAs are located in the 3’ untranslated regions or in the noncoding transcript of a specific gene (19). For example, circBIRC6 acts as a sponge for miR-34a and miR-145 to abate the downregulation of these target genes and restrain the differentiation of human embryonic stem cells (20). CircITCH promotes the expression of ITCH and CBL by sponging miR-214 and miR-22-3p (18). Second, circRNAs can serve as decoys, transporters, or scaffolds to regulate cellular function by binding to proteins (21). CircRNAs have binding sites on RNA-binding proteins (RBPs) and can recruit RBPs. For example, circPABPN1 binds to HuR to suppress the translation of the linear form of PABPN1 mRNA (22). Additionally, circ-Foxo3 acts as a protein scaffold to suppress cell cycle progression by binding to the cell cycle proteins cyclin-dependent kinase 2 and cyclin-dependent kinase inhibitor 1 (23). Third, several studies have provided interesting evidence that circRNAs can be translated (24–26). Translated circRNAs use the start codon of a hosting mRNA and have evolutionary conserved termination codons, which are unique to the circular open reading frame. Because of their closed structure, circRNAs have been demonstrated to undergo cap-independent translation given the existence of the internal ribosome entry sites (25). For example, circ-ZNF609 contains an open reading frame, and heat shock induces intense and meaningful activation of the translation of the flagged circ-ZNF609 (26). Proteins produced by circMbl3 have also been detected. Circ-ZNF609 and circMbl3 confirm the protein-coding potential of circRNAs and may change the view that circRNAs have previously considered ncRNAs. Fourth, circRNAs are signaling molecules related to the intercellular communication. The exosomes, derived from multivesicular bodies, are secreted by most cells and are the vesicles with a diameter of 30-200 nm (27). CircRNAs are more abundant in the exosomes than their linear counterparts and are sorted to the exosomes by changing the corresponding miRNA levels in the host cells. For example, the level of circHIPK3 is higher in the exosomes derived from U-2 OS cells than the level of linear HIPK3. In addition, circRNAs in the exosomes remain circular and are stable in the serum. Some circRNAs that retain their biological activity in the exosomes can be transferred to the recipient cells to regulate their function (28, 29). For example, CDR1as circRNA in the exosomes relieves miR-7-induced growth suppression in the recipient cells. The emergence of circRNAs in the exosomes may indicate their pivotal role in cellular and organ crosstalk considering the functions of the exosomes in mediating the communications between the cells.

Current progress in the studies of circRNAs revealed several functions of circRNAs; however, additional studies are needed to detect the relationships between circRNAs and human diseases.

## Role of circRNAs in T1DM

T1DM, also known as autoimmune diabetes mellitus, is characterized by irreversible destruction of insulin-producing β-cells in pancreatic islets (30) and an absolute paucity of endogenous insulin with subsequent hyperglycemia (31). Genetics and environmental factors are two critical pathogenic elements that contribute to the onset and development of T1DM (32, 33). Screening for islet-specific autoantibodies is the main tool to confirm and predict T1DM (34). However, this approach cannot fully meet the needs for early prediction and prevention of T1DM. CircRNAs may identify individuals at high risk of developing T1DM and help to elucidate the pathogenesis of T1DM (**Table 1**).

**Table 1:** Role of circRNAs in DM and associated complications.

Disease	CircRNA	Expression level	Finding	References
**T1DM**	CircARHGAP12	Down	May be a potential biomarker for T1DM	(5)
	CircRNA 000286	Down	May participate in proinflammatory cytokine-mediated β-cell dysfunction	(35)
	CircRNA 017277	Down	May participate in proinflammatory cytokine-mediated β-cell dysfunction	(35)
	Hsa_circ_0060450	Up	Functions as a sponge of miR-199a-5p to restrain the JAK-STAT signaling pathway and further inhibit macrophage-mediated inflammation	(36)
	CircPPM1F	Up	Regulates M1 macrophage activation *via* the circPPM1F−HuR−PPM1F−NF-κB axis	(37)
	Hsa_circ_0002202	Up	Mediates IFN-I-induced macrophage inflammation	(38)
	Hsa_circ_0002473	Up	Associates with a pro-apoptotic feedback loop in β-cells	(39)
	Hsa_circ_0072697	Down	May mediate the risk for T1DM *via* the circ_0072697−miR-15a−UBASH3A network	(40, 41)
**T2DM**	CircHIPK3	Down Up	Regulates the expression level of pivotal β-cell genes in rats May function as a potential biomarker for the diagnosis of T2DM in patients	(5) (42)
	CiRS-7	Down Up	Silencing ciRS-7 diminishes prolactin-stimulated proliferation of primary rat β-cells May function as a potential biomarker for the diagnosis of T2DM in patients	(5) (42)
	Rno_circRNA_008565	Down	Binds with rno-miR-504 to influence autophagy of rat islet β-cells	(43)
	Circ-Tulp4	Down	Regulates β-cell proliferation *via* miR-7222-3p−soat1−cyclin D1 signaling	(44)
	Novel_circ_0016196	Down	Interacts with miR-877-3p and modulates mitochondrial apoptosis	(45)
	Novel_circ_0016198	Down	Interacts with miR-877-3p and modulates mitochondrial apoptosis	(45)
	Novel_circ_0005686	Down	Interacts with miR-877-3p and modulates mitochondrial apoptosis	(45)
	Hsa_circ_0002590	Up	Interacts with miR-149-5p and inhibits insulin secretion	(45)
	Novel_circ_000372	Up	Interacts with miR-149-5p and inhibits insulin secretion	(45)
	Hsa-circRNA11783-2	Down	May be a sponge for miR-608 and miR-3907	(46)
	Circ_CCNB1	Up	May be related to the progression of T2DM and may function as a potential biomarker for the diagnosis of T2DM	(47)
	Circ_0009024	Down	May be related to the progression of T2DM and may function as a potential biomarker for the diagnosis of T2DM	(47)
	Hsa_circ_0071106	Up	May be used as a diagnostic marker for T2DM	(48)
	Hsa_circ_0063425	Down	Binds with miR-19a-3p and is positively related to *AKT*	(49)
	Hsa_circ_0056891	Down	Binds with miR-1-3p and is positively related to *PI3K*	(49)
	CircGlis3	Up	Impairs β-cell function under lipotoxic conditions and may be a potential biomarker for T2DM	(50)
	CircANKRD36	Up	Correlates with chronic inflammation in T2DM; silencing of circANKRD36 suppresses insulin resistance and inflammation by targeting miR-145 *via* XBP1	(51, 52)
	Hsa_circ_0054633	Up	May be used as a marker for T2DM	(53, 54)
**GDM**	Circ_0008285	Up	Correlates with total cholesterol	(55)
	Circ_0001173	Down	Correlates with glycated hemoglobin	(55)
	Hsa_circ_102682	Down	Is valuable for diagnosis of GDM	(56)
	CircVEGFC	Up	Has high sensitivity and specificity in early diagnosis of GDM and is positively related to fetal malformations and hypertension	(57)
	CircACTR2	Up	Is related to higher rates of premature delivery, miscarriage, intrauterine death, fetal malformation, intrauterine infection, and hypertension, but is not associated with macrosomia and intrauterine distress	(58)
	Hsa_circRNA_0039480	Up	Has a diagnostic value in the first, second, and third trimesters of pregnancy	(59)
	Circ-PNPT1	Up	Facilitates HG-induced biological dysfunction in trophoblasts cell *via* the miR-889-3p−PAK1 axis	(60)
	Hsa_circ_0005243	Down	Mediates trophoblast dysfunction and inflammation by regulating the β-catenin and NF-κB signaling pathways	(61)
	Circ_0074673	Up	Regulates the proliferation, migration, and angiogenesis of HG-HUVECs *via* the miR-1200−MEOX2 axis	(62)
	Hsa_circ_0054633	Up	Is useful for GDM diagnosis and plays a role in the regulation of β-cell proliferation and glucose metabolism	(63, 64)
	Hsa_circRNA_102893	Down	Is valuable for diagnosis of GDM	(65, 66)
**DR**	Circ-ITCH	Down	Modulates angiogenesis by sponging miR-22	(67)
	Hsa_circ_0002570	UP	Promotes angiogenesis and inflammation in hRMECs *via* the circ_0002570−miR-1243−angiomotin axis	(68)
	Circ_0005015	UP	Facilitates angiogenesis by the circ_0005015−miR-519d-3p−MMP-2/XIAP/STAT3 pathway	(69)
	Hsa_circ_0081108	UP	Facilitates angiogenesis by the hsa_circ_0081108−miR-29b−VEGF axis	(70)
	Circular RNA-ZNF609	UP	Facilitates angiogenesis by the miR-615-5p−MEF2A pathway	(71)
	Mmu_circ_0000254	Up	Mediates retinal vascular dysfunction and functions as a sponge of miR-579	(72)
	CircDNMT3B	Down	Mediates vascular dysfunction in diabetic retinas by the circDNMT3B−miR-20b-5p−BAMBI axis	(73)
	Circular RNA-ZNF532	UP	Regulates DM-induced retinal pericyte degeneration by sponging miR-29a-3p	(74)
	Hsa_circRNA_063981	Up	May be a potential biomarker for DR	(75)
	Hsa_circRNA_404457	Up	May be a potential biomarker for DR	(75)
	Hsa_circRNA_100750	Up	May be a potential biomarker for DR	(75)
	Hsa_circRNA_406918	Up	May be a potential biomarker for DR	(75)
	Hsa_circRNA_104387	Up	May be a potential biomarker for DR	(75)
	Hsa_circRNA_103410	Up	May be a potential biomarker for DR	(75)
	Hsa_circRNA_100192	Up	May be a potential biomarker for DR	(75)
	Hsa_circ_0001953	Up	Has a diagnostic value in PDR patients	(76)
	CircEhmt1	Up	Reduces HG-induced apoptosis and inflammation *via* the NFIA/NLRP3 pathway	(77)
	Hsa_circ_0041795	UP	Promotes HG-triggered cell apoptosis *via* the miR-646−VEGFC pathway	(78)
	CircRNA_0084043	UP	Promotes HG-triggered cell apoptosis *via* the circRNA_0084043−miR-140-3p−TGFA axis	(79)
	Circ_001209	UP	Promotes HG-triggered cell apoptosis *via* the miR-15b-5p−COL12A1 axis	(80)
	Circ-PSEN1	UP	Mediates ferroptosis of HG-treated retinal pigment epithelial cells *via* the miR-200b-3p−cofilin-2 axis	(81)
	CircHIPK3	Down	Mediates retinal vascular dysfunction *via* the circHIPK3−miR-30a-3p−VEGFC/WNT2/FZD4 axis	(82, 83)
**DN**	Circ_0123996	Up	Facilitates fibrosis by sponging miR-149-5p	(84)
	CircEIF4G2	Up	Facilitates fibrosis *via* the circEIF4G2−miR-218−SERBP1 pathway	(85)
	CircRNA_15698	Up	Facilitates fibrosis *via* the circRNA_15698−miR‐185−TGF‐β1 axis	(86)
	Circ_0000491	Up	Facilitates fibrosis *via* the circRNA_0000491−miR‐101b−TGFβRI axis	(87)
	CircLRP6	Up	Mediates mesangial cell dysfunction *via* the circLRP6−miR‐205−HMGB1−TLR4/NF‐κB regulatory network	(88)
	CircHIPK3	Up	Promotes cell proliferation and fibrosis by sponging miR-185	(89)
	Circ_DLGAP4	Up	Promotes cell proliferation and fibrosis *via* the circ_DLGAP4−miR-143−ERBB3/NF-κB/MMP-2 axis	(90)
	Hsa_circ_0125310	Up	Promotes cell proliferation and fibrosis *via* the circ_0125310−miR‐422a−IGF1R-p38 axis	(91)
	CircACTR2	Up	Promotes inflammation and fibrosis in HK-2 cells	(92)
	Circ-FBXW12	Up	Promotes human mesangial cell inflammation and ECM accumulation *via* the miR-31-5p−LIN28B axis	(93)
	Hsa_circ_0004442	Up	Promotes human mesangial cell inflammation and ECM accumulation *via* the hsa_circ_0004442−miR-126-5p/miR-204-5p−AKT/NF-κB pathway	(94)
	Circ_0080425	Up	Inhibits cell proliferation and fibrosis *via* the circ_0080425−miR-24-3p−FGF11 axis	(95)
	Circ_LARP4	Down	Inhibits cell proliferation and fibrosis by sponging miR-424	(96)
	Circ-AKT3	Down	Inhibits fibrosis *via* the circ-AKT3−miR-296-3p−E-cadherin pathway	(97)
	CircSMAD4	Down	Inhibits fibrosis *via* the circSMAD4−miR-377-3p−*BMP7* pathway	(98)
	Circ-ITCH	Down	Alleviates renal inflammation and fibrosis in STZ-induced diabetic mice by regulating the miR-33a-5p−SIRT6 axis	(99)
	CircRNA_010383	Down	Knockdown facilitates proteinuria and renal fibrosis in DN; is a sponge for miRNA-135a	(100)
	Hsa_circ_0003928	Up	Regulates HG-induced inflammation *via* the miR-151-3p−Anxa2 axis	(101)
	Hsa_circRNA_102682	Down	May be relevant to the pathogenesis of hyperhomocysteinemia in DN and may be a potential biomarker for DN	(102)
	Circ_0000712	Up	Promotes apoptosis *via* the circ_0000712−miR-879-5p−SOX6 axis	(103)
	Circ_WBSCR17	Up	Promotes apoptosis *via* the miR-185-5p−SOX6 regulatory axis	(104)
	Circ_0000285	Up	Promotes apoptosis *via* the circ_0000285−miR-654-3p−MAPK6 axis	(105)
	Mmu_circRNA_0000309	Down	Is involved in germacrone-mediated improvement of DN *via* the miR-188-3p−GPX4 axis	(106)
	Circ_0037128	Up	Promotes cell proliferation and fibrosis *via* the circ_0037128−miR-17-3p−AKT3 axis; promotes inflammation and fibrosis in HK-2 cells *via* the circ_0037128−microRNA-497-5p−NFAT5 axis	(107, 108)
	Circ_0000064	Up	Promotes cell proliferation and fibrosis by sponging miR-143; is involved in emodin-mediated alleviation of DN *via* the circ_0000064 −miR-30c-5p−Lmp7 axis	(109, 110)
**DC**	CircRNA11783-2	Down	May be correlated with DC	(46)
	CircRNA_000203	Up	Promotes myocardial fibrosis *via* the circRNA_000203−miR-26b-5p−Col1a2/CTGF axis	(111)
	CircRNA_010567	Up	Promotes myocardial fibrosis *via* the circRNA_010567−miR-141−TGF-β1 axis	(112)
	CircHIPK3	Up	Promotes myocardial fibrosis *via* the circHIPK3−miR-29b-3p−*Col1a1*−*Col3a1* axis	(113)
	Hsa_circ_0076631	Up	Promotes an inflammatory response and pyroptosis *via* the hsa_circ_0076631−miR-214-3p−caspase-1 axis	(114)
	Circ_0071269	Up	Promotes an inflammatory response and pyroptosis *via* the microRNA-145−gasdermin A axis	(115)
**DF**	Hsa_circ_0084443	Up	Modulates keratinocyte migration *via* the PI3K, EGFR, and ERK signaling pathways	(116)
	Hsa_circ_0000907	Up	May function as a novel biomarker for early diagnosis of DFU	(117)
	Hsa_circ_0057362	Up	May function as a novel biomarker for early diagnosis of DFU	(117)
	CircRNA_072697	Up	May be a new target for the treatment of DFU	(118, 119)
**Diabetic neuropathy**	CircHIPK3	Up	Functions as a sponge for miR-124, and silencing this circRNA eases mechanical hyperalgesia and thermal hyperalgesia	(120)

Increasing evidence has shown that circRNAs participate in the onset and development of T1DM by various mechanisms. First, circRNAs are involved in inflammation. A study *in vivo* identified 1,020 upregulated and 902 downregulated circRNAs in mouse pancreatic β-cells stimulated by the cytokines interferon (IFN)-γ, interleukin (IL)-1β, and tumor necrosis factor (TNF)-α. These differentially expressed circRNAs include circRNAs 000286 and 017277 whose downregulation reduces insulin biosynthesis and secretion and facilitates apoptosis of pancreatic β-cells (35), indicating that circRNAs may participate in proinflammatory cytokine-mediated β-cell dysfunction. Second, circRNAs may be associated with T1DM by influencing macrophages, which contribute to the loss of β-cells and subsequent development of hyperglycemia and are involved in the pathogenesis of T1DM (121, 122). For example, hsa_circ_0060450, circPPM1F, and hsa_circ_0002202 are upregulated in peripheral blood mononuclear cells (PBMCs) from T1DM patients and sponge miR-199a-5p to inhibit the JAK-STAT signaling pathway induced by type I IFN (IFN-I) to further inhibit macrophage-mediated inflammation (36), regulate M1 macrophage activation *via* the circPPM1F−HuR−PPM1F−NF-κB axis (37), and mediate IFN-I-induced macrophage inflammation (38), respectively. However, additional *in vivo* experiments are needed to draw stronger conclusions concerning hsa_circ_0060450 and hsa_circ_0002202.

Given that autoantigens are generated as the consequence of β-cell death (123), islet autoantibodies cannot identify high-risk individuals before β-cell dysfunction is manifested. Thus, other early biomarkers are necessary. CircARHGAP12 is decreased in the islets of NOD mice, which is a mouse model of spontaneously developing T1DM (5). Altered expression level of circARHGAP12 under diabetic conditions suggested that this circRNA may be a potential biomarker for T1DM. Another study detected 8,214 circRNAs in human plasma by microarray analysis of the samples of three subjects with new-onset T1DM and identified 61 upregulated and 7 downregulated circRNAs. Notably, the results of gene analysis revealed that these differentially expressed circRNAs participate in inflammation and the pathogenesis of T1DM (9), supporting the future applications of circRNAs as novel biomarkers of T1DM. Additionally, hsa_circ_0072697 is significantly downregulated in patients with T1DM (40). This circRNA regulates the expression of *UBASH3A via* the circ_0072697−miR-15a−UBASH3A network. Two T1DM-associated variants of *UBASH3A*, including the risk alleles at rs11203203 and rs80054410, can increase *BASH3A* expression in human primary CD4^+^ T cells (41) that mediate the destruction of β-cells (33), indicating that circ_0072697 may mediate the effects of a risk factor for T1DM *via* UBASH3A and may serve as a biomarker of T1DM.

Presently, insulin injection is required daily for most T1DM patients. Insulin can maintain blood glucose at an appropriate level but is not a radical treatment. Hence, a new and effective therapy is essential, and circRNAs may offer these possibilities. For example, hsa_circ_0002473, which originates from DNAJ homolog subfamily C member 3 (DNAJC3), is upregulated in T1DM patients. DNAJC3 promotes the translocation of the 78-kDa glucose-regulated protein (GRP78) to the plasma membrane to trigger a proapoptotic feedback loop in β-cells (39). This axis may provide novel therapeutic ideas for T1DM because blocking GRP78 is expected to decrease apoptosis to preserve β-cells (40).

In summary, circRNAs may be involved in T1DM *via* various pathways, such as inflammation and macrophages, can be considered biomarkers, and may provide new targets to treat T1DM.

## Role of circRNAs in T2DM

T2DM is characterized by insulin resistance and relative insulin deficiency and accounts for 90 to 95% of adult DM cases (124); T2DM prevalence in children and adolescents has increased globally in the past several decades (125). The expansion of T2DM prevalence from the adult population to people of all ages has propelled this disease to become one of the greatest public health burdens in the world. Therefore, an in-depth understanding of T2DM is valuable.

CircRNAs are correlated with T2DM (**Table 1**) and may participate in T2DM pathogenesis. For example, rno_circRNA_008565 is downregulated in a T2DM rat model and binds rno-miR-504 to influence autophagy in rat islet β-cells (43). Additionally, the level of circ-Tulp4 is decreased in pancreatic islets of T2DM mice, and a subsequent study demonstrated that circ-Tulp4 regulates β-cell proliferation *via* miR-7222-3p−soat1−cyclin D1 signaling (44). Another study using a T2DM rat model induced by streptozotocin (STZ) showed that circANKRD36 expression is higher in T2DM rats, and silencing circANKRD36 decreases blood glucose and suppresses insulin resistance and inflammation by targeting miR-145−XBP1 (52). In addition to the results of the studies in rats, some circRNAs have been identified to be related to T2DM in patients. Recently, a study identified 21 differentially expressed circRNAs, including 5 upregulated and 16 downregulated circRNAs, in venous blood of fasting T2DM patients. Further analysis demonstrated that downregulated circRNAs (novel_circ_0016196, novel_circ_0016198, and novel_circ_0005686) interact with miR-877-3p to modulate mitochondrial apoptosis, and upregulated circRNAs (hsa_circ_0002590 and novel_circ_000372) interact with miR-149-5p to inhibit insulin secretion (45). Another study detected a significant downregulation of hsa-circRNA11783-2 in peripheral blood in the T2DM group, and this circRNA has a notable correlation with T2DM. Hsa-circRNA11783-2 sponges miR-608 and miR-3907; however, the relationships between these two miRNAs and T2DM need further studies (46). Additionally, circRNAs may be involved in T2DM due to an association with IL-6 and TNF-α, which are strongly correlated with T2DM (126). For example, the level of circANKRD36 in peripheral blood leukocytes is elevated in T2DM patients and positively correlates with the level of IL-6 (51). Another study highlighted that circ_CCNB1 is clearly upregulated and positively associated with IL-6 and TNF-α, whereas circ_0009024 is downregulated and negatively associated with IL-6 and TNF-α in the serum samples of T2DM patients (47). Overall, circRNAs may be related to T2DM *via* the inflammation-associated pathways.

The exact time of the onset of T2DM is usually challenging to determine. Delayed diagnosis may increase the risk of the development of DM-associated complications. Therefore, precise early identification of T2DM is valuable. Evidence has shown that circRNAs may function as potential biomarkers of T2DM. A study using RNA sequencing detected 683 differentially expressed circRNAs in the blood samples of T2DM patients, including 354 upregulated and 329 downregulated circRNAs. The results of functional analysis suggested that these differentially expressed circRNAs may be involved in the apoptosis pathway (10), indicating possible use of circRNAs as biomarkers in the near future. Additionally, receiver operating characteristic (ROC) curves are usually used to estimate the ability of circRNAs to distinguish T2DM patients from healthy controls. Hsa_circ_0071106 is upregulated in the T2DM group and may increase the risk of T2DM by sponging hsa-miR-29a-5p. The area under the ROC curve for this circRNA was 0.690 (48). Additionally, the areas under the curve (AUCs) for hsa_circ_CCNB1 and circ_0009024 were 0.9255 and 0.9592, respectively (47). Recently, circHIPK3 and ciRS-7 were shown to be significantly increased in the peripheral blood samples of T2DM patients and prediabetic individuals, respectively. CircHIPK3 may be able to distinguish T2DM patients from control subjects (AUC=0.665), and ciRS-7 may distinguish prediabetic patients from healthy control subjects (AUC=0.605) (42). Furthermore, the results of a retrospective case−control study in enrolled T2DM patients and control subjects indicated that hsa_circ_0054633 is gradually increased from the baseline condition to the prediabetic state and to actual T2DM in the corresponding groups. The AUCs for this circRNA for the diagnosis of prediabetes and T2DM were 0.747 and 0.72, respectively (53). Additionally, impaired fasting glucose (IFG) indicates a prediabetic status, and evaluation of IFG is vital to relieve the DM burden. Recent evidence has shown that a panel of 2 circRNAs, hsa_circ_0063425 and hsa_circ_0056891, may be able to distinguish T2DM and IFG patients from healthy controls given that the corresponding AUCs were 0.873 and 0.719 for T2DM and IFG, respectively. Moreover, hsa_circ_0063425, which sponges miR-19a-3p, and hsa_circ_0056891, which sponges miR-1-3p, are positively related to *AKT* and *PI3K*, respectively, suggesting that these circRNAs may influence PI3K/AKT signaling to modulate the pathogenesis of T2DM (49). These results suggest that circRNAs may function as potential biomarkers for the diagnosis of T2DM. Additionally, lipotoxicity contributes to insulin resistance and β-cell apoptosis (50). The expression of β-cell-derived exosomal circGlis3 is increased under lipotoxic conditions; circGlis3 may be transferred to islet endothelial cells to reduce the viability, migration, and angiogenesis of these cells. Moreover, an increase in exosomal circGlis3 was detected in the serum in the mouse models of DM and in individuals with T2DM. Since the serum is an easily accessible biological fluid, exosomal circGlis3 may be a potential biomarker for T2DM (50). Moreover, insulin treatment may relieve inflammation in T2DM patients. Serum hsa_circ_0054633, which is positively correlated with TNF-α and IL-17, is downregulated in the T2DM with insulin treatment group compared with that in the groups of healthy adults and T2DM patients without insulin treatment, suggesting that hsa_circ_0054633 may be a potential early indicator of the anti-inflammatory effects of insulin treatment (54).

**Figure 3:**
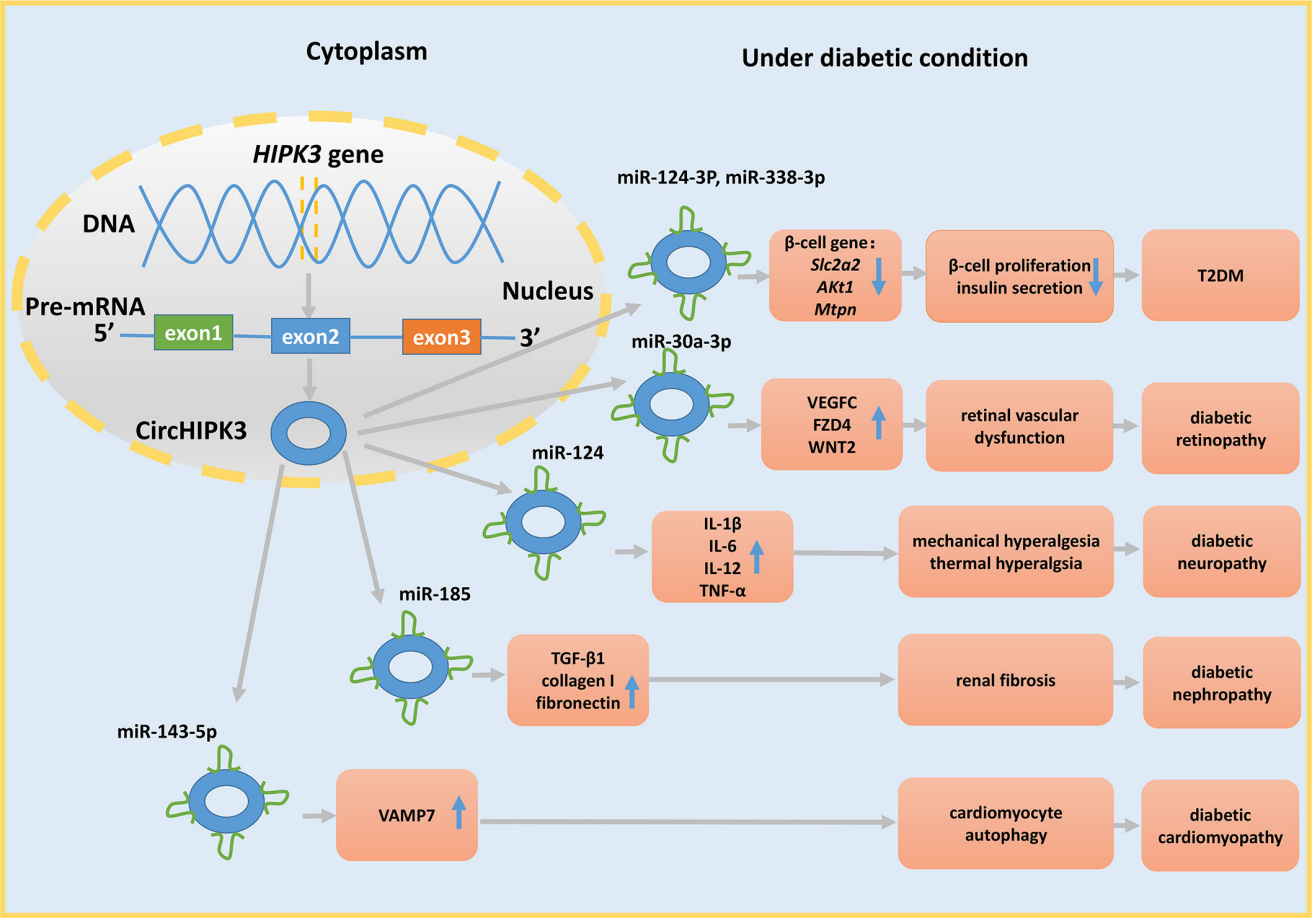
The miRNA sponge role of circHIPK3 in DM and its complications. CircHIPK3 is derived from exon 2 of the HIPK3 gene and acts as a sponge for various miRNAs to regulate cell function, further contributing to the development of DM and its complications.

CircRNAs may have potential therapeutic functions in T2DM. A decrease in the expression level of ciRS-7 in the islets of mice causes a reduction in insulin secretion. Furthermore, silencing ciRS-7 diminishes prolactin-stimulated proliferation of primary rat β-cells (5). Silencing circHIPK3 causes decreased insulin secretion and production in response to glucose and decreases β-cell proliferation. Moreover, circHIPK3 regulates the expression level of pivotal β-cell genes, such as *Slc2a2, Akt1*, and *Mtpn*, by sponging miR-124-3p and miR-338-3p (**Figure 3**) (5). These results indicate that a drug targeting circHIPK3 and/or ciRS-7 to maintain the appropriate levels of these circRNAs may help to avoid β-cell dysfunction and abnormal insulin secretion.

These studies provide evidence that circRNAs may be involved in the pathogenesis, may be biomarkers for T2DM, and may even represent a therapy for T2DM; however, considerable work is needed to evaluate their safety and efficacy before these circRNAs can be widely applied in clinical practice.

## Role of circRNAs in GDM

GDM is manifested as impaired glucose tolerance and elevated fasting glucose levels during pregnancy and normal blood glucose levels before pregnancy. The incidence rate of GDM is increasing with prevalence of obesity (127). Unless appropriate treatment is provided, GDM will cause adverse pregnancy outcomes for the offspring and mother (128). The emerging role of circRNAs in GDM has drawn public attention (**Table 1**).

CircRNAs may participate in GDM progression considering associations between these circRNAs and clinical indicators in GDM patients. For example, hsa_circ_0054633 expression is positively associated with 2-h glucose, glycated hemoglobin (GHBA1), and GHBA1c (63) and was speculated to play a role in the regulation of β-cell proliferation and glucose metabolism during pregnancy because this circRNA was reported to be involved in the progression of the cell cycle and molecular catabolism (64). Hsa_circRNA_102893 is downregulated in the plasma of pregnant women with GDM and acts as a sponge for miR-33a-5p and miR-197-3p (65). Hsa_circRNA_102893 is likely to be related to GDM because miR-33a is positively associated with total serum cholesterol and low-density lipoprotein cholesterol in DM patients (66), and miR-197-3p is correlated with impaired glycemic control (129). Similar to this study, another recent study indicated that circ_0008285 expression is upregulated and circ_0001173 is downregulated in the plasma specimens of GDM patients; the former circRNA is notably correlated with total cholesterol, and the latter circRNA is correlated with glycated hemoglobin (55). A retrospective case–control study revealed that hsa_circ_102682 is downregulated in the case group and significantly correlates with triglycerides, apolipoprotein A1 (APOA1), APOB, and 1-h blood glucose in the serum of GDM patients (56). In addition to the correlations with clinical indicators, circRNAs are also related to multiple adverse events of GDM. Recently, circular RNA vascular endothelial growth factor C (circVEGFC) was identified as a novel regulator of glucose metabolism, and its level was shown to be increased in GDM patients on the day of admission and 1 month before and after delivery. This circRNA is also positively associated with fetal malformation and hypertension (57). Similarly, circRNA actin-related protein 2 homolog (circACTR2) is increased in GDM patients and is associated with higher rates of premature delivery, miscarriage, intrauterine death, fetal malformation, intrauterine infection, and hypertension (58). However, these results were derived from the association studies; therefore, additional *in vivo* and *in vitro* experiments will be indispensable to verify the relationships between circRNAs and GDM, leading to more definitive conclusions.

Fetal exposure to maternal diabetes is related to a higher lifetime risk of obesity and development of T2DM in the offspring, possibly contributing to the worldwide diabetes epidemic (130). Thus, it is essential to distinguish GDM patients from healthy individuals early. A study demonstrated that 229 circRNAs are significantly upregulated and 278 circRNAs are significantly downregulated in the umbilical cord blood exosomes of GDM patients, indicating that circRNAs may be the sentinels of GDM (131). Additionally, hsa_circRNA_0054633 expression is elevated in the blood during the second and third trimesters of pregnancy and may be a potential biomarker for GDM (AUC=0.793) (63). Hsa_circRNA_0039480 encapsulated in the exosomes is upregulated in the GDM group and has a diagnostic value in the first, second, and third trimesters of pregnancy (AUC = 0.704, AUC = 0.898, and AUC = 0.698, respectively) (59). Hsa_circRNA_102893 and hsa_circ_102682 are also valuable for the diagnosis of GDM with AUCs of 0.806 and 0.684, respectively (56, 65). The AUC of circVEGFC on the day of admission was 0.8927, indicating a higher sensitivity and specificity of this circRNA for early diagnosis of GDM (57).

CircRNAs may have a possible for GDM treatment based on their influence on the cell functions. For example, circ-PNPT1 expression is higher in placental tissues from individuals with GDM and trophoblasts treated with high glucose (HG). A further study demonstrated that circ-PNPT1 facilitates a biological dysfunction of HG-induced trophoblasts *via* the miR-889-3p−PAK1 axis (60). Hence, interference with this signaling pathway may maintain normal trophoblast function, implying a promising targeted therapy for GDM. Hsa_circ_0005243 is decreased in both the placenta and plasma of GDM patients. Cell proliferation and migration are inhibited after hsa_circ_0005243 depletion, which causes a trophoblast dysfunction and inflammation *via* the β-catenin and nuclear factor (NF)-κB pathways (61). Additionally, exosomal circ_0074673 is increased in the exosomes from GDM patients and in human umbilical vein endothelial cells (HUVECs) cocultured with the exosomes. Silencing circ_0074673 promotes the proliferation, migration, and angiogenesis of HG-HUVECs *via* the miR-1200−MEOX2 axis (62). In summary, a drug that maintains normal function of the cells by regulating circRNA expression levels may open new avenues for GDM therapy.

Overall, abundant evidence suggests a crucial role of circRNAs in the pathogenesis of GDM and indicates that circRNAs may be considered biomarkers and may provide potential therapies for GDM.

## Role of circRNAs in DM-associated complications

DM-related complications can be classified as acute and chronic. The IDF estimated that more than four million adults aged 20–79 years died because of DM and its associated complications in 2019 (132). Therefore, a thorough understanding of DM-related complications is required because of their enormous social burden (**Table 1**).

### DR

DR includes proliferative DR (PDR) and nonproliferative DR (NPDR); DR remains a primary cause of blindness globally, along with vascular leakage, inflammation, and angiogenesis (133). CircRNAs may be related to the pathogenesis of DR in various ways. First, some circRNAs influence DR by serving as stimulators or inhibitors of neovascularization and may thus have a certain pathogenic importance for the progression of DR (134). For example, circ-ITCH is downregulated in retinal pigment epithelial cells isolated from diabetic rats. This circRNA reduces angiogenesis by sponging miR-22 because its overexpression inhibits an increase in matrix metalloproteinase (MMP)-2 and MMP-9, which are positive regulators of neovascularization (67). Conversely, a recent study demonstrated that hsa_circ_0002570 is dramatically increased in the retinal proliferative fibrovascular membranes from DR patients and in human retinal microvascular endothelial cells (hRMECs), and knockdown of this circRNA inhibits HG-induced angiogenesis and inflammation in hRMECs *via* the circ_0002570−miR-1243−angiomotin axis (68). Circ_0005015 and hsa_circ_0081108 are upregulated in the plasma of DR patients and HG-induced hRMECs, respectively. The results of functional analysis showed that both these circRNAs facilitate angiogenesis by regulating the proliferation and migration of endothelial cell *via* the circ_0005015−miR-519d-3p−MMP-2/XIAP/STAT3 pathway (69) and the hsa_circ_0081108−miR-29b−VEGF axis (70), respectively. Circular RNA-ZNF609 expression is elevated in DM mice, and silencing this circRNA reduces retinal vessel loss and inhibits pathological angiogenesis in DM mice *via* the miR-615-5p−MEF2A pathway (71).

Second, circRNAs regulate DR by influencing pericyte loss, microaneurysms, and acellular microvasculature, which are the crucial characteristics of diabetic retinas (135). For example, mmu_circ_0000254 is upregulated and sponges miR-579. Silencing of this circRNA increases retinal vascular leakage and inflammation and the levels of acellular vasculature, pericyte loss, and microaneurysms in diabetic retinas (72). In contrast, silencing circHIPK3 decreases the number of retinal acellular capillaries, vascular leakage, and inflammation *via* the circHIPK3−miR-30a-3p−VEGFC/WNT2/FZD4 axis (**Figure 3**). CircHIPK3 derived from exon 2 of the *HIPK3* gene (136) influences retinal endothelial cells (82). The level of this circRNA is markedly decreased in diabetic retinas and retinal endothelial cells. *In vitro* studies demonstrated that the changes in circHIPK3 expression affect the viability, proliferation, migration, and tube formation in retinal endothelial cells (83). Additionally, circDNMT3B is downregulated in hRMECs, and circDNMT3B overexpression reduces the number of acellular capillaries in diabetic rats *via* the circDNMT3B−miR-20b-5p−BAMBI axis (73). A very recent study reported that circular RNA-ZNF532 is upregulated in the retinal vessels in pericytes under diabetic stress, and silencing cZNF532 reduces the viability, proliferation, and differentiation of pericytes and aggravates retinal pericyte degeneration by sponging miR-29a-3p (74).

Considering the severity of DR, improved biomarkers are urgently needed for early identification of DR patients. The levels of seven circRNAs, including hsa_circRNA_063981, hsa_circRNA_404457, hsa_circRNA_100750, hsa_circRNA_406918, hsa_circRNA_104387, hsa_circRNA_103410, and hsa_circRNA_100192, are distinctly increased in the serum of T2DM patients with PDR (75), implying a potential role of circRNAs as biomarkers for DR. Additionally, hsa_circ_0001953 was reported to be increased in the blood of PDR patients and is positively correlated with the duration of DM and hemoglobin A1c levels. This study indicated a potential value of hsa_circ_0001953 in distinguishing PDR patients from NPDR patients and healthy controls (76).

CircRNAs have been shown to be the potential targets for treatment of DR due to their involvement in the regulation of cell death. For example, exosomal circEhmt1 released from hypoxia-pretreated pericytes reduces HG-induced apoptosis and inflammation in cultured endotheliocytes and protects endotheliocytes against HG-induced injury *in vitro via* the NFIA/NLRP3 pathway (77). Hsa_circ_0041795 and circRNA_0084043 are clearly upregulated in HG-treated retinal pigment epithelial cells, and circ_001209 is upregulated in HG-treated human retinal vascular endothelial cells. All these circRNAs are able to promote HG-triggered apoptosis of the cells by the miR-646−VEGFC pathway (78), circRNA_0084043−miR-140-3p−TGFA axis (79), and miR-15b-5p−COL12A1 axis (80), respectively. Additionally, ferroptosis is a form of programmed cell death involved in DR. For example, circ-PSEN1 is upregulated in HG-treated ARPE19 cells and its inhibition mitigates ferroptosis of HG-treated retinal pigment epithelial cells *via* the miR-200b-3p−cofilin-2 axis (81). Thus, a new treatment for DR needs to maintain an appropriate expression level of circRNAs to normalize the properties of the cells.

### DN

DN is a severe complication in DM patients. Dysfunction of mesangial cells and podocytes accelerates the development of DN, causing end-stage renal disease and representing a massive healthcare burden (137). Accumulating evidence has revealed that DN is significantly associated with circRNAs.

Fibrosis and inflammation are essential characteristics of DN (138). Circ_0123996 expression is increased in the mouse models of DN, and the expression of fibrosis-related proteins is alleviated when the level of circ_0123996 is decreased. Additionally, circ_0123996 acts as a sponge of miR-149-5p to participate in DN (84). The levels of circEIF4G2, circRNA_15698, and circ_0000491 are dramatically elevated in the kidney of db/db mice *in vivo* and in a DN cellular model *in vitro*. Knockdown of these circRNAs restrains the expression of collagen type I and fibronectin, and these circRNAs are able to promote fibrosis *via* the circEIF4G2−miR-218−SERBP1 pathway (85), circRNA_15698−miR‐185−TGF‐β1 axis (86), and circRNA_0000491−miR-101b−TGFβRI axis (87), respectively. The level of circLRP6 is increased in HG-treated mesangial cells, and this circRNA acts as a sponge for miR-205 to target high mobility group box 1 (HMGB1), which facilitates HG-induced proliferation, oxidative stress, extracellular matrix (ECM) accumulation, and inflammation in mesangial cells *via* the toll-like receptor 4 (TLR4)/NF-κB pathway. Further studies demonstrated that circLRP6 knockdown increases the expression of miR‐205 and reduces the expression of HMGB1 mRNA and protein, protecting mesangial cells from dysfunction *via* the circLRP6−miR‐205−HMGB1−TLR4/NF‐κB regulatory network (88). Recent studies showed that the levels of circ_0037128, circ_0000064, and circHIPK3 (**Figure 3**) are notably elevated in mesangial cells treated with HG, and circ_DLGAP4 and circ_0125310 are also elevated in the exosomes isolated from HG-treated mesangial cells. All these circRNAs promote mesangial cell proliferation and aggravate fibrosis *via* the circ_0037128−miR-17-3p−AKT3 axis (107), sponging miR-143 (109), sponging miR-185 (89), the circ_DLGAP4−miR-143−ERBB3/NF-κB/MMP-2 axis (90), and the circ_0125310−miR‐422a−IGF1R-p38 axis (91), respectively. Similarly, two *in vitro* studies using HG-induced HK-2 cells demonstrated that circACTR2 (92) and circ_0037128 (108) are increased and that silencing these circRNAs decreases inflammation and fibrosis in HK-2 cells. Circ_0037128 regulates DN *via* the circ_0037128−microRNA-497-5p−nuclear factor of activated T cells 5 (NFAT5) axis. Additionally, circ-FBXW12 and hsa_circ_0004442 are upregulated in the sera of DN patients and HG-induced human mesangial cells, and their silencing inhibits HG-induced inflammation and ECM accumulation in human mesangial cells *via* the miR-31-5p−LIN28B axis (93) and hsa_circ_0004442−miR-126-5p/miR-204-5p−AKT/NF-κB pathway (94), respectively.

The functions of certain circRNAs associated with DN are opposite to the functions of circRNAs listed above. Circ_0080425 is upregulated in DN mice, and circ_LARP4 is downregulated in a DN cellular model; both these circRNAs inhibit cell proliferation and fibrosis *via* the circ_0080425−miR-24-3p−FGF11 axis (95) and sponging miR-424 (96), respectively. Circ-AKT3 and circSMAD4 are clearly decreased in DN mice and in mouse mesangial cells treated with HG. These circRNAs inhibit fibrosis *via* the circ-AKT3−miR-296-3p−E-cadherin (97) and circSMAD4−miR-377-3p−*BMP7* pathways (98), respectively. The expression of circ-ITCH is clearly decreased in HG-treated rat mesangial cells. Further experiments demonstrated that circ-ITCH alleviates renal inflammation and fibrosis in STZ-induced diabetic mice by regulating the miR-33a-5p−SIRT6 axis (99). Additionally, circRNA_010383 is dramatically reduced in diabetic kidneys, mesangial cells, and tubular epithelial cells stimulated under the HG conditions, and knockdown of this circRNA facilitates proteinuria and renal fibrosis in DN by serving as a sponge for miRNA-135a (100). Additionally, the expression of hsa_circ_0003928 is upregulated in the blood samples of DN patients, and an increase in hsa_circ_0003928 expression alleviates inflammation in HK-2 cells *via* the miR-151-3p−Anxa2 axis (101).

CircRNAs may be biomarkers. For example, hsa_circRNA_102682 is markedly downregulated in the serum of diabetic patients with high levels of homocysteine, which is an independent risk factor for DN (102), indicating that circRNAs may be associated with the pathogenesis of hyperhomocysteinemia in DN and may be potential biomarkers for DN.

CircRNAs may play a role in DN treatment considering relationships between circRNAs and cell apoptosis. For example, circ_0000712, circ_WBSCR17, and circ_0000285 are increased in DN mice and the cellular models of DN and promote HG-induced apoptosis in DN *via* the circ_0000712−miR-879-5p−SOX6 axis (103), circ_WBSCR17−miR-185-5p−SOX6 regulatory axis (104), and circ_0000285−miR-654-3p−mitogen-activated protein kinase 6 (MAPK6) axis (105), respectively. These results imply a possible circRNA-targeted therapy for DN by altering circRNA levels. Furthermore, circRNAs regulate the effect of other drugs. For example, germacrone alleviates kidney damage and suppresses podocyte apoptosis in a DN model in mice. The expression of mmu_circRNA_0000309 is decreased in DN mice, and its knockdown mediates drug resistance to germacrone by abolishing the antiapoptotic and anti-injury effects of germacrone *via* the mmu_circRNA_0000309−miR-188-3p−GPX4 axis (106). Additionally, emodin exerts a protective effect in DN *via* the circ_0000064−miR-30c-5p−Lmp7 axis (110).

### DC

DC is characterized by cardiac dysfunction. The pathological mechanism of DC includes myocardial fibrosis, dysfunctional remodeling, and associated diastolic dysfunction, leading to heart failure (139).

Recent studies demonstrated the associations between DC and circRNAs. For example, the expression of hsa-circRNA11783-2 is reduced in both the T2DM and coronary artery disease groups (46), indirectly implying that hsa-circRNA11783-2 may be correlated with DC. However, the specific mechanism of participation of circRNA11783-2 in DC is poorly understood and requires further investigation. Additionally, circRNA_000203, circRNA_010567, and circHIPK3 are increased in diabetic mice. These circRNAs promote myocardial fibrosis *via* the circRNA_000203−miR-26b-5p−Col1a2/CTGF axis (111), circRNA_010567−miR-141−TGF-β1 axis (112), and circHIPK3−miR-29b-3p−*Col1a1*−*Col3a1* axis (**Figure 3**) (113), respectively, and these data elucidate the pathogenesis of DC.

Suppression of cell death and inflammation is considered an important therapy for DC (140). Pyroptosis is a type of programmed cell death associated with inflammation (141). CircRNAs may be used as a novel treatment of DC. Hsa_circ_0076631 is increased in HG-treated cardiomyocytes and in the serum of patients with DM, and circ_0071269 is upregulated in HG-treated H9c2 cells. Both these circRNAs promote the inflammatory response and pyroptosis *via* the hsa_circ_0076631−miR-214-3p−caspase-1 axis (114) and microRNA-145−gasdermin A axis (115), respectively. Knockdown of these circRNAs protects against diabetic cardiomyopathy injury, implying an accurate therapy for DC.

### DF

Diabetic foot (DF) is a severe diabetic complication, and diabetic foot ulceration (DFU) contributes to high mortality of DM. Therefore, an understanding of the molecular mechanism of DFU is urgently needed.

A study demonstrated that hsa_circ_0084443 located in the cytoplasm of human epidermal keratinocytes is increased in the DFU samples and decreases the migration capacity of keratinocytes *via* the PI3K, EGFR, and ERK signaling pathways (116), indicating that circRNAs are involved in DFU.

Another study identified 10 upregulated and 23 downregulated circRNAs significantly different between patients with DF and DM and patients with DM alone (142), indicating that circRNAs may be the biomarkers for DF. Additionally, the expression levels of hsa_circ_0000907 and hsa_circ_0057362 are high in the serum of patients with DF, and the AUCs for serum hsa_circ_0000907 and hsa_circ_0057362 in diagnosis of early DFU are 0.9389 and 0.8792, respectively, implying that these circRNAs may be used to identify DFU (117).

Recent evidence has shown that the level of circRNA_072697, which acts as a sponge of miR-3150a-3p, is increased in DFU. The level of circRNA_072697 is related to miR-3150a-3p *via* the MAPK signaling pathway (118). Given that p38 MAPK is a member of the MAPK family and that its inhibition relieves chronic wounds associated with DM (119), circRNA_072697 may be a new target for treatment of DFU.

### Diabetic neuropathy

DN-related pain is manifested in approximately 30% of T2DM patients and increases with the duration of DM. CircHIPK3 is not only involved in DR, DN, DC, and T2DM but is also upregulated in the serum of DM patients with neuropathic pain and in the dorsal root ganglia of STZ-induced DM rats. Furthermore, a higher grade of neuropathic pain in T2DM patients is associated with greater concentrations of circHIPK3 in the serum. Further experiments revealed that circHIPK3 acts as a sponge for miR-124, and silencing this circRNA decreases the expression levels of IL-1β, IL-6, IL-12, and TNF-α to ease mechanical and thermal hyperalgesia (**Figure 3**). Moreover, a study demonstrated that the treatment with intrathecal circHIPK3 shRNA alleviates neuropathic pain in diabetic rats (120). However, additional extensive research is necessary to apply this therapy to patients in an early stage of the disease.

In summary, regardless of human sample analysis or experiments in the cells or even animals, evidence indicates that circRNAs mediate DM-associated complications *via* various regulatory networks and may be the biomarkers and potential therapeutic targets for DM-associated complications.

## Conclusion

DM is a chronic serious metabolic disease. T1DM, T2DM, and GDM are three common types of DM. Impaired glucose tolerance and impaired fasting glucose indicate a prediabetic state. Prediabetes is a condition in which individuals have impaired glucose metabolism but do not meet the diagnostic criteria of DM; prediabetes is positively associated with the risk of all-cause mortality and incidence of cardiovascular outcomes, stroke, chronic kidney disease, cancer, and dementia (143). Intermediate states of hyperglycemia are becoming an increasing problem, bringing major global challenges. The etiology of DM remains unclear. Therefore, individuals at high risk of DM must be identified early. Currently, despite the advancements in medical technology, many individuals develop DM and its complications, resulting in not only physical harm but also mental effects and even huge economic burden for individuals and countries. Therefore, novel biomarkers and novel treatments are required to overcome DM.

CircRNAs are abundant and stable transcripts, emerging as the key regulators of gene expression. Steady advances in RNA sequencing technology resulted in identification of many circRNAs. Accumulating evidence has demonstrated that circRNAs are related to the onset and progression of DM and its complications. Under pathological conditions, the expression levels of circRNAs are upregulated or downregulated. Differential expression of circRNAs between the diabetic and normal physiological conditions indicates a link between circRNAs and DM. CircRNAs play three roles under diabetic conditions: participation in DM pathogenesis, application as potential biomarkers, and the use as potential therapeutic targets. First, circRNAs contribute to the progression of DM by influencing cellular phenotypes, such as fibrosis and proliferation, and cellular functions, such as insulin secretion, *via* various signaling pathways. Second, circRNAs are able to distinguish DM patients from healthy control subjects, and diagnostic abilities of circRNAs have been demonstrated by ROC analysis. However, some circRNAs alone do not have high sensitivity and specificity, and a combination of two or more circRNAs may enhance their diagnostic value. Third, the influence of altered expression of circRNAs on disease conditions and the role of circRNAs in regenerative medicine imply that circRNAs may be used as novel agents for treatment of DM. CircRNAs have particular benefits for diagnosis and therapy, such as (1) stability (2), relatively long half-lives in the cells, and (3) availability of circRNAs in the body fluids, such as blood.

However, many hurdles for clinical application of circRNAs exist. First, evidence of the roles of circRNAs in DM is inadequate and needs to be verified by many experiments *in vivo* and *in vitro*. Second, the dynamics, pharmacokinetics, and toxic effects of circRNAs need to be further evaluated.

In conclusion, circRNAs are involved in DM and help us gain a better and deeper understanding of the mechanism of DM and its complications, although many challenges remain in the field.

## Author contributions

WF searched references, wrote the first draft of the paper, and revised the text. HP, ZX, and ZZ critically revised the text and provided substantial scientific contributions. GH proposed the project and revised the manuscript. All authors approved the final version of the manuscript.

## Funding

This study was supported by the National Key R&D Program of China (2018YFC1315600, 2018YFC1315603), National Science and Technology Major Project (2020ZX09201-028) and the National Natural Science Foundation of China (81820108007, 81800745)

## Conflict of interest

The authors declare that the research was conducted in the absence of any commercial or financial relationships that could be construed as a potential conflict of interest.

## Publisher’s note

All claims expressed in this article are solely those of the authors and do not necessarily represent those of their affiliated organizations, or those of the publisher, the editors and the reviewers. Any product that may be evaluated in this article, or claim that may be made by its manufacturer, is not guaranteed or endorsed by the publisher.
